# Recognizing a Forgotten Disease: A Case Report of Tetanus in an Unvaccinated Adolescent

**DOI:** 10.7759/cureus.116370

**Published:** 2026-09-16

**Authors:** Jordan Scott, Michael Reihart

**Affiliations:** 1 Emergency Medicine, Liberty University College of Osteopathic Medicine, Lynchburg, USA; 2 Emergency Medicine, Penn State Lancaster Medical Center, Lancaster, USA

**Keywords:** amish, neuromuscular rigidity, pediatric infectious disease, tetanus, trismus, vaccination

## Abstract

Tetanus is a rare but potentially life-threatening vaccine-preventable disease in the United States. Because tetanus is now rarely encountered in the United States, diagnosis may be delayed despite classic clinical findings.

We report the case of an unvaccinated sixteen-year-old Amish male who presented with progressive trismus and cervical rigidity thirteen days after tympanoplasty with mastoidectomy and incus debridement. Initial symptoms were attributed to a postoperative infectious process and treated with oral antibiotics. Progressive jaw stiffness and neck rigidity prompted emergency evaluation. Physical examination demonstrated severe trismus with approximately 1 cm maximal mouth opening and marked cervical muscle rigidity. Laboratory studies and CT (computerized tomography) imaging were unrevealing. A presumptive clinical diagnosis of tetanus was made based on characteristic findings and complete absence of prior immunization. Treatment with tetanus immune globulin, metronidazole, benzodiazepines, supportive care, and active immunization resulted in complete recovery.

This case highlights the continued importance of vaccination history, recognition of classic clinical manifestations, and maintenance of diagnostic vigilance for rare vaccine-preventable diseases.

## Introduction

Tetanus is caused by tetanospasmin, a potent neurotoxin produced by Clostridium tetani [1,2]. Although routine vaccination has dramatically reduced disease incidence in the United States, approximately 30 cases continue to be reported annually, particularly among unvaccinated or inadequately immunized individuals [3]. Mortality remains significant despite advances in critical care [1,3]. Because tetanus is now rarely encountered in developed countries, continued clinical vigilance is important.

The diagnosis remains primarily clinical because laboratory studies are neither sensitive nor specific [2,3]. Recognition therefore depends on identification of characteristic findings including trismus, muscle rigidity, painful spasms, and an appropriate epidemiologic history [1,2]. We present a case of tetanus in an unvaccinated adolescent following ear surgery, in whom the initial symptoms were attributed to a postoperative infectious process, highlighting the diagnostic challenge of this presentation and the importance of vaccination status in recognizing tetanus.

## Case presentation

A 16-year-old Amish male with congenital conductive hearing loss underwent elective left tympanoplasty, mastoidectomy, and incus debridement. He had never received routine childhood immunizations.

Eleven days after surgery, he developed progressive neck pain and stiffness and was prescribed amoxicillin for presumed otitis media. Despite treatment, symptoms continued to worsen over the following two days. He subsequently presented to the emergency department with increasing difficulty opening his mouth and marked neck rigidity.

Physical examination demonstrated severe trismus with approximately 1 cm maximal incisor separation, rigidity of the anterior cervical and trapezius musculature, and several superficial abrasions of the lower extremities without evidence of cellulitis. He was afebrile and hemodynamically stable. Neurologic examination was otherwise unremarkable.

Laboratory evaluation demonstrated no significant abnormalities, as summarized in Table 1. Computed tomography of the head and neck revealed expected postoperative findings without evidence of abscess formation or deep neck space infection.

**Table 1:** Laboratory findings at emergency department presentation. WBC: White blood cell count; Hgb: Hemoglobin; Na: Sodium; K: Potassium; Cl: Chloride; CO₂: Carbon dioxide; Cr: Creatinine; µL: Microliter; g/dL: Grams per deciliter; mmol/L: Millimoles per liter; mg/dL: Milligrams per deciliter.

Laboratory Test	Patient Value	Standard Reference Range
WBC	5.08 × 10³/µL	4.5–11.0 × 10³/µL
Hemoglobin	13.5 g/dL	13.0–16.0 g/dL
Sodium	137 mmol/L	135–145 mmol/L
Potassium	4.0 mmol/L	3.5–5.0 mmol/L
Chloride	102 mmol/L	98–106 mmol/L
CO₂ / Bicarbonate	27 mmol/L	22–29 mmol/L
Creatinine	0.6 mg/dL	0.5–1.0 mg/dL
Glucose	91 mg/dL	70–100 mg/dL

The combination of progressive trismus, neck rigidity, lower extremity abrasions, and complete absence of vaccination history raised strong suspicion for tetanus. Differential diagnoses included peritonsillar abscess, retropharyngeal abscess, meningitis, temporomandibular joint pathology, and medication-induced dystonia. Following administration of ceftriaxone and tetanus-containing vaccine at the referring facility, the patient was transferred to a tertiary pediatric center for definitive management because tetanus immune globulin was unavailable locally.

Definitive treatment included human tetanus immune globulin, intravenous metronidazole, diazepam for muscle rigidity, wound management, supportive care, and initiation of active immunization. The patient remained hemodynamically stable and did not develop generalized spasms or autonomic instability. Symptoms improved progressively over 72 hours.

He was discharged on hospital day three with oral medications and close follow-up. At one-month follow-up, trismus and neck rigidity had completely resolved, and he had resumed normal activities. The chronological progression of the patient’s clinical course is summarized in Table 2.

**Table 2:** Chronological timeline of the patient’s clinical course. ED: Emergency department; PICU: Pediatric intensive care unit; TIG: Tetanus immune globulin; IV: Intravenous; Tdap: Tetanus, diphtheria, and acellular pertussis vaccine.

Time	Clinical course
Day 0	Left tympanoplasty, mastoidectomy, and incus debridement
Day 11	Developed neck pain; diagnosed with otitis media and prescribed amoxicillin
Day 13	Presented to ED with progressive neck pain, trismus, and rigidity; received ceftriaxone and Tdap and was transferred
Days 13–16	PICU admission; treated with TIG, IV metronidazole, and IV diazepam with improvement
Day 16	Discharged on oral metronidazole and diazepam
Approximately one month later	Gradual resolution of symptoms

## Discussion

This case demonstrates the continued relevance of tetanus in populations with lower vaccination uptake. Although uncommon in the United States, tetanus should remain within the differential diagnosis for patients presenting with trismus and unexplained muscle rigidity, particularly when vaccination history is incomplete or absent [3].

Tetanospasmin enters peripheral motor neurons and undergoes retrograde axonal transport to the central nervous system. There it blocks release of gamma-aminobutyric acid and glycine from inhibitory interneurons, resulting in unopposed muscle contraction, rigidity, and spasms [2]. The incubation period generally ranges from one to twenty-one days, consistent with the timeline observed in this patient [3].

Trismus is among the earliest and most recognizable manifestations of tetanus [2]. Progression from facial and jaw involvement to cervical musculature reflects the characteristic descending pattern of disease [2]. Overall, this patient’s presentation was consistent with commonly reported features of tetanus, particularly progressive trismus and cervical muscle rigidity with preserved sensorium, although the absence of generalized spasms or autonomic instability represented a comparatively limited clinical course [1,2]. Importantly, laboratory testing and imaging are frequently normal and are primarily useful for excluding alternative diagnoses rather than confirming tetanus [2,3].

The differential diagnosis of trismus and neck rigidity is broad. Deep neck space infections often present with fever, systemic illness, and imaging abnormalities. In this patient, the absence of fever or systemic illness, together with unrevealing imaging, made a deep neck space infection less likely. Meningitis typically produces fever, headache, altered mental status, or cerebrospinal fluid abnormalities. Acute dystonic reactions are generally associated with dopamine-blocking medications and abrupt symptom onset. In contrast, tetanus classically presents with progressive rigidity, preserved sensorium, and a compatible wound or exposure history [2].

Management consists of neutralization of unbound toxin with tetanus immune globulin, antimicrobial therapy and appropriate wound care, control of muscle spasms, airway and supportive management when necessary, monitoring for autonomic dysfunction, and active immunization because tetanus disease does not confer protective immunity [1,2,4,5]. Key principles of tetanus management are summarized in Table 3 [4,5].

**Table 3:** Key principles of tetanus management. TIG: Tetanus immune globulin; IU: International units; IM: intramuscular; IV: Intravenous; PO: Oral. Management recommendations, including TIG administration, antimicrobial therapy, wound management, control of muscle spasms, airway and supportive care, monitoring for autonomic dysfunction, and active immunization, are based on World Health Organization guidance [4] and represent general treatment recommendations rather than the specific doses administered to this patient.

Management Domain	Recommended Management
Toxin neutralization	Human tetanus immune globulin (TIG), 500 IU, administered as soon as possible
Antimicrobial therapy	Metronidazole 500 mg IV or PO every 6 hours; penicillin G is an alternative
Wound management	Clean wounds and debride as indicated
Muscle spasm control	Benzodiazepines are preferred
Airway/respiratory management	Maintain a safe airway; provide mechanical ventilation when clinically necessary
Autonomic dysfunction	Monitor for and manage autonomic dysfunction
Active immunization	Administer an age-appropriate tetanus toxoid-containing vaccine because tetanus disease does not confer immunity
Supportive care	Hospitalization and supportive care, including minimizing stimuli that can precipitate reflex spasms

Early diagnosis is essential because progression to generalized tetanus can result in respiratory failure, autonomic instability, prolonged hospitalization, and death [1,2].

The most important diagnostic clue in this case was the complete absence of prior immunization. Recognition of this epidemiologic risk factor substantially narrowed the differential diagnosis and facilitated timely treatment. Clinicians caring for patients from communities with lower vaccination rates should maintain awareness of vaccine-preventable diseases that may be encountered infrequently in contemporary practice [5,6].

## Conclusions

Tetanus remains an uncommon but clinically important diagnosis, particularly in patients who are unvaccinated or incompletely immunized. Progressive trismus and cervical rigidity should prompt immediate consideration of tetanus even when laboratory and imaging findings are unrevealing. Because diagnosis is primarily clinical, careful assessment of vaccination history and recognition of characteristic neuromuscular findings are essential to avoiding diagnostic delay. Early treatment with tetanus immune globulin, antimicrobial therapy, control of muscle rigidity and spasms, appropriate wound management, supportive care, and active immunization is recommended to reduce morbidity and the risk of progression to severe disease. This case underscores the importance of maintaining clinical vigilance for vaccine-preventable diseases even when they are rarely encountered in contemporary practice.

## References

[1] Thwaites CL, Loan HT (2015). Eradication of tetanus. Br Med Bull.

[2] Cook TM, Protheroe RT, Handel JM (2001). Tetanus: A review of the literature. Br J Anaesth.

[3] Hughes MM, Amin AB, Rubis AB (2026). Tetanus surveillance - United States, 2009-2023. MMWR Surveill Summ.

[4] World Health Organization (2010). Current recommendations for treatment of tetanus during humanitarian emergencies. https://www.who.int/publications/i/item/current-recommendations-for-treatment-of-tetanus-during-humanitarian-emergencies.

[5] Kettunen C, Nemecek J, Wenger O (2017). Evaluation of low immunization coverage among the Amish population in rural Ohio. Am J Infect Control.

[6] Yoder JS, Dworkin MS (2006). Vaccination usage among an old-order Amish community in Illinois. Pediatr Infect Dis J.

